# Multi-Directional Crosswalk of the Harris Hip Score and the Hip Disability and Osteoarthritis Outcome Score

**DOI:** 10.3390/jcm14051432

**Published:** 2025-02-20

**Authors:** Chan Hee Cho, Kerry Costi, Deepti Sharma, Dominic Thewlis, Lucian B. Solomon, Stuart A. Callary

**Affiliations:** 1 Faculty of Health and Medical Sciences, Centre for Orthopaedic and Trauma Research, The University of Adelaide, Adelaide, SA 5005, Australia; chanhee.cho@adelaide.edu.au (C.H.C.); deepti.sharma@sa.gov.au (D.S.); dominic.thewlis@adelaide.edu.au (D.T.); bogdan.solomon@sa.gov.au (L.B.S.); stuart.callary@adelaide.edu.au (S.A.C.); 2 Department of Orthopaedics and Trauma, Royal Adelaide Hospital, Adelaide, SA 5000, Australia

**Keywords:** hip arthroplasty, hip replacement, PROMS, patient-reported outcome measures, crosswalks, mapping, linking

## Abstract

**Background:** Despite the popularity of the modified Harris Hip Score (mHHS) to monitor patient-reported outcome measures (PROMs) following Total Hip Arthroplasty (THA) over the last 5 decades, International Joint Registries have recently favoured the Hip disability and Osteoarthritis Outcome Score (HOOS). The ability to convert mHHS collected in historical and ongoing studies would be beneficial to benchmark more recent HOOS reports. Hence, this study aimed to create multi-directional crosswalks between mHHS and HOOS. **Methods:** Forty-nine patients undergoing primary THA prospectively completed both HHS and HOOS forms pre-operatively and at either 3, 6 and/or 12 months postoperatively. The Equipercentile (EQ) and Linear Regression (LR) crosswalk methodology were used. The Mean Absolute Error (MAE) of the crosswalk-derived scores was established against patient-derived (PD) scores. **Results:** There was a strong correlation between PD mHHS and HOOS (0.90) and HOOS-12 (0.90). The MAE of mHHS-to-HOOS-12 crosswalk was 10.4 (EQ) and 10.1 (LR). Subcategory activity had a larger contribution towards the error in the crosswalks than pain. **Conclusions:** This is the first crosswalk to facilitate conversion of mHHS and HOOS scores, which are required in long-term THA quality-assurance and research studies, which often span 2 decades of expected implant survivorship.

## 1. Introduction

Osteoarthritis (OA), a severe degenerative joint disease, causes chronic pain, affects mobility, and reduces quality of life in individuals over the age of 40 [1]. The prevalence of OA worldwide has increased from 4.8% in 1990 to 7.6% in 2020, and with increasing age, it is estimated that there will be 62.6 million individuals with hip OA [1]. Older age, occupation, gender, obesity, joint anatomy abnormalities, and previous trauma or surgeries can predispose the joint to osteoarthritic changes [2]. Total Hip Arthroplasty (THA) is an effective surgical procedure to relieve pain and improve function for patients [3,4,5] with OA. However, it has been reported that THA may yield suboptimal results for up to 25% of patients, and that post-operative pain and functional limitations last up to 1 or 2 years [6]. Patient-Reported Outcome Measures (PROMs) have been used extensively to monitor pain, mobility, and quality of life following THA using questionnaires pre- and post-surgery. The PROM outcomes collated have been used for a multitude of quality assurance and research studies that benefit patients and healthcare providers by enabling comparison of outcomes of different surgical techniques and rehabilitation protocols. PROMs also allow patient feedback on treatment and increase patient awareness of expected outcomes, ensuring a high-level standard of treatment management.

A consensus document published in the 1990s 3 decades ago recommended numerous PROMs to measure and standardise THA outcomes [7]. The Harris Hip Score (HHS) remains the most commonly utilised outcome measure for hip function and symptoms despite being introduced in 1969 [8]. It is important to note that the HHS is not a PROM as we consider it today. The HHS outcome measure provides a score out of 100 that is both patient- (91) and doctor-derived (9). More recently, PROMs using self-reporting questionnaires are preferred as they can be administered directly to the patient remotely without surgeon influence. A modified HHS (mHHS) was introduced for this use that excludes the doctor-reported range of motion score out of 9 [9,10,11]. More recently, newer self-reporting PROMs, including the Hip Disability and Osteoarthritis Outcome Score (HOOS) [12], have been incorporated into national hip registries. Additionally, a simplified 12-question version of the original HOOS (HOOS-12) was developed in 2019 [13]. However, at many national centres, the use of the HOOS and HOOS-12 has inadvertedly overlapped long-standing prospective hospital-based PROM collections. The ability to longitudinally monitor PROMs is necessary due to the long-term survivorship of THA implants now exceeding 2 decades, but it is optimal to minimise the patient burden of completing questionnaires regularly during the postoperative period.

To facilitate meaningful comparisons between PROMs, a crosswalk methodology has been used [14,15,16]. A multitude of methods in the literature have been used to create PROM crosswalks. Some of these include the Equipercentile Method, regression models (general linear regression equating, Tobit models, ordinary least squares, and quantile regression), item response theory equating, and Bayesian probability models [16,17,18]. There is no consensus on which mapping method should be used to create a crosswalk between two PROMs [17,19], although the Equipercentile Method (EQ) and the Linear Regression method (LR) methods are the most commonly used for arthroplasty outcome measures [16]. Studies have created either uni-directional or multi-directional PROMs where unidirectional PROMs enable the conversion of scores from one specific PROM to another, while multidirectional PROMs enable conversion between the scores in either direction. A recent systematic review has identified 17 studies describing 35 crosswalks for converting PROMs following knee and hip arthroplasty [16]. Within the review, there were 12 crosswalks created between two PROMs following THA. Only five crosswalks included the conversion of HOOS scores [17,20,21,22,23], and no study established crosswalks of mHHS with any other PROM. Therefore, the aim of this study was to create multi-directional crosswalks that enable comparisons between the HHS and the HOOS in primary THA patients.

## 2. Materials and Methods

**Figure 1:**
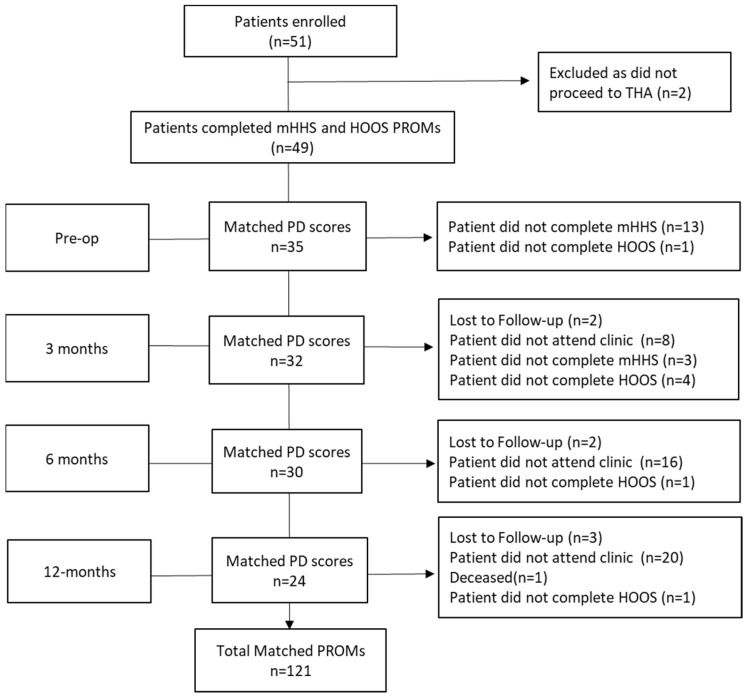
Flowchart of number of matched patient-derived PROMs collected at each time point.

To derive the crosswalk, we included data from a previous prospective study, which recruited patients scheduled to undergo primary THA at the Royal Adelaide Hospital (Adelaide, Australia) between August 2016 and February 2018 [24,25]. Patients were recruited and included in the study if the primary reasons for THA were OA, avascular necrosis of the femoral head, or inflammatory osteoarthritis. Of the initial 51 patients, 49 patients completed the HOOS and mHHS questionnaires prospectively pre-operatively, and at 3, 6, and 12 months post-operatively (Figure 1). Patients completing the HHS and HOOS questionnaires at the same time points were included in this study. Patients completed both questionnaires independently at scheduled visits. The mean age of patients was 63 (range 23–86), and there were 27 males and 22 females. There were 42 patients with OA, six with AVN, and one with juvenile arthritis. This study was approved by our local human research ethics committee (CALHN ethics #010310a and #R20160807). The consent form to participate in the study was distributed to all participants and signed.

The mHHS is made up of nine variables (weighted accordingly). The domains consist of pain, function, and absence of deformity. The mHHS has a maximum of 91 points (pain—44 points, function—47 points, range of motion—5 points, and deformity—4 points) [8]. The question regarding public transport (1 point) is also not applicable as it is not used in the same manner as it had been when the HHS was developed. Therefore, the possible maximum score is out of 90 [9]. Within the function component of the total score, activities of daily living are worth 14 points (climbing stairs, wearing shoes/socks, sitting), and gait is worth 33 points. A higher mHHS indicates less dysfunction.

The HOOS consists of 40 question items that assess five different subcategories: pain (10 questions), symptoms and stiffness (5 questions), activities of daily living (17 questions), function in sports and recreational activities (4 questions), and quality of life (4 questions). All questions are scored from 0 to 4, and each of the subcategories is calculated as the sum of the items included. The HOOS dimensions are then transformed into a 0–100 scale (0 worst, 100 best). The HOOS-12 scores were extracted from the completed HOOS questionnaires. The overall score of the HOOS-12 also is scored from 0–100 scale (0 worst, 100 best).

To determine the necessary sample size required to detect a correlation between HHS and HOOS, a power analysis for correlation tests was conducted using the pwr package in R Studio [26]. The minimum correlation required between PD scores of 0.3 was based on Dorans et al. [27], with a significance level of 0.05 and a power of 0.8. Under these conditions, the required minimum sample size is 67 matched PROM scores.

Both EQ and LR methodology were used to create multi-directional crosswalks between mHHS and HOOS and the mHHS and HOOS-12. The EQ equating method is a non-parametric approach and involves calculating the percentile rank of the raw PD scores. Scores with the same percentile rank were considered equivalent [28]. The LR equating method assumes there is a linear relationship between the PD scores of each PROM and uses the derived linear equation to create the crosswalk [28]. The validity of the crosswalks was assessed by evaluating the Mean Absolute Error (MAE) of the Crosswalk-Derived (CWD) scores against Patient-Derived (PD) scores [17]. The greater the difference between PD scores from the CWD indicates a larger error present in the crosswalk. The total scores for the mHHS are out of 90, and for the HOOS and HOOS-12, they are out of 100. The MAE is presented in relation to the total scores. Subcategory analysis of CWD HOOS and mHHS Pain and Activity scores were investigated to identify potential larger sources of error using the EQ method as both pain scores are not continuous but gradual. HOOS had 10 questions investigating patient pain (40 points) and 17 questions investigating patient activity (68 points). HOOS-12 has 4 questions on patient pain (16 points) and 8 questions investigating patient activity (12 points). Both HOOS and HOOS-12 pain and activity scores were converted into scores ranging from (0–100 points). The mHHS had 1 question on patient pain (44 points) and 3 questions on activities of daily living (stairs, shoes, and sitting) (13 points).

### Statistical Analysis

Statistical analysis was performed in GraphPad Prism (version 9.1.1). To be able to create the crosswalks, a Spearman’s correlation coefficient (R) of 0.3 or higher between the PD scores of the two PROMs is required [27]. To establish the crosswalk validity, CWD scores were compared against PD scores to establish the MAE.

## 3. Results

Of the 51 patients enrolled, 49 patients completed both HHS and HOOS pre-operatively, at 3, 6, and 12 months. There were a total of 121 matching HOOS and HHS scores at the same time points that were used to create the crosswalks (35 scores at pre-op, 32 scores at 3 months, 30 scores at 6 months, and 24 scores at 12 months) (Figure 1).

**Figure A1:**
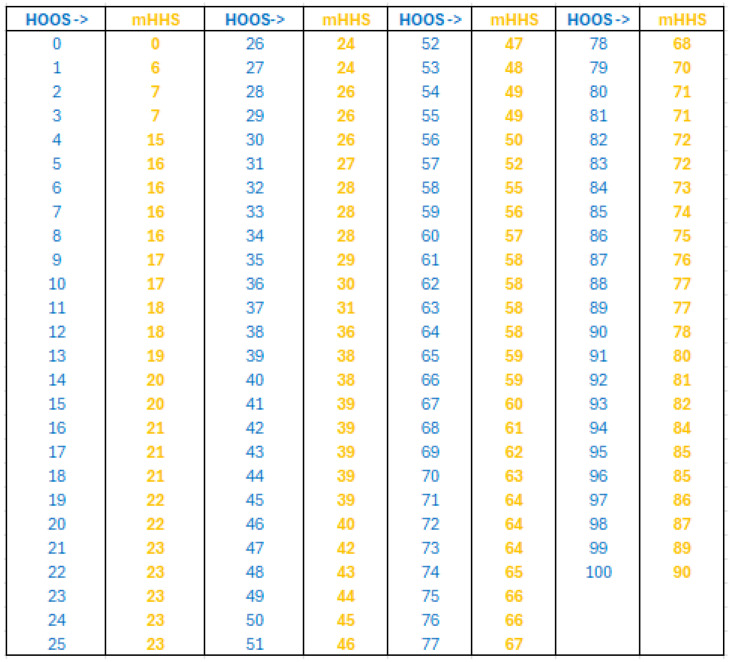
HOOS-to-mHHS crosswalk made by the Equipercentile Method (EQ).

**Figure A2:**
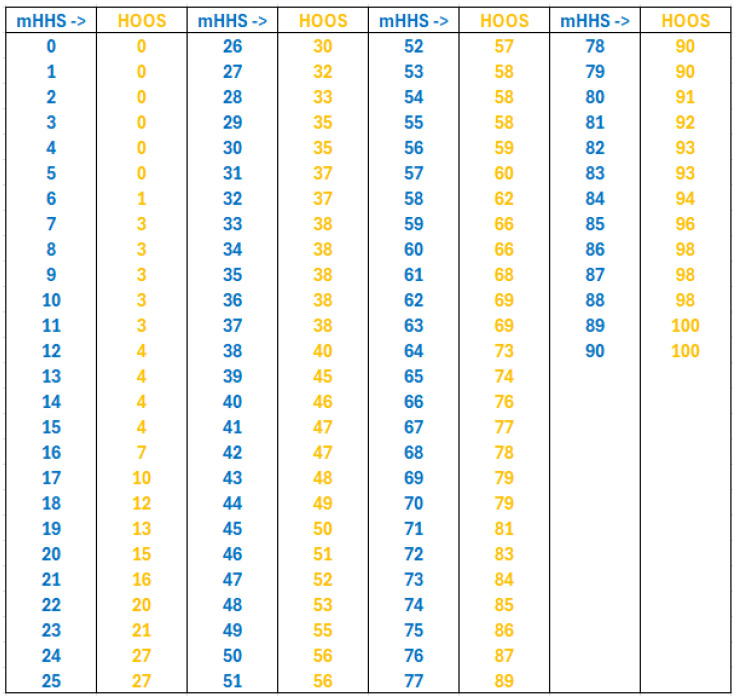
mHHS-to-HOOS crosswalk made by the Equipercentile Method (EQ).

**Figure A3:**
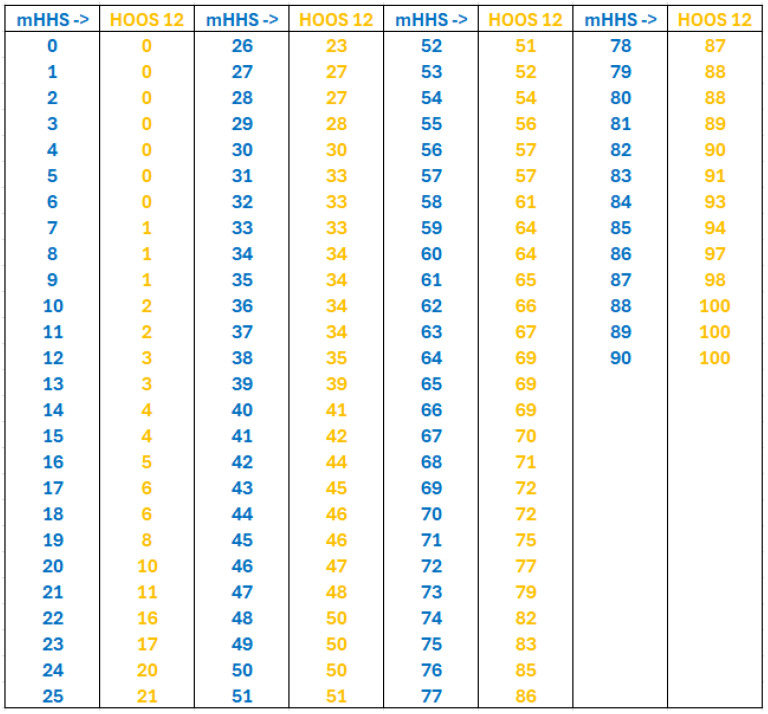
HOOS-12-to-mHHS crosswalk made by the Equipercentile Method (EQ).

**Figure A4:**
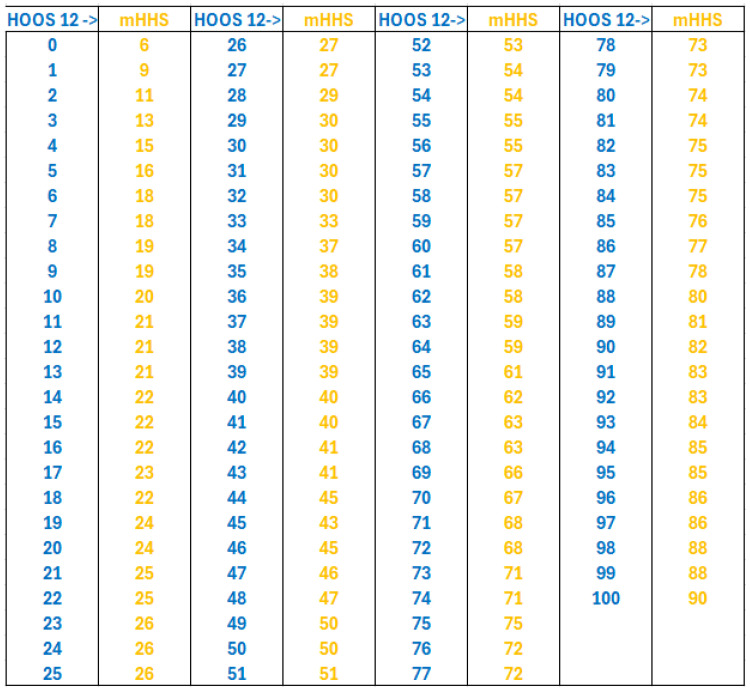
mHHS to HOOS-12 crosswalk made by the Equipercentile Method (EQ).

**Figure A5:**
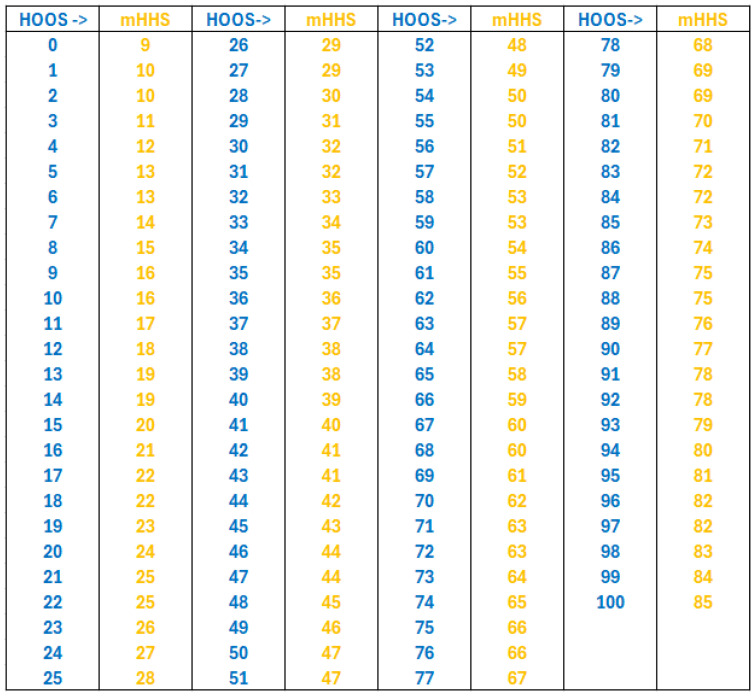
HOOS-to-mHHS crosswalk made by the Linear Regression Method (LR).

**Figure A6:**
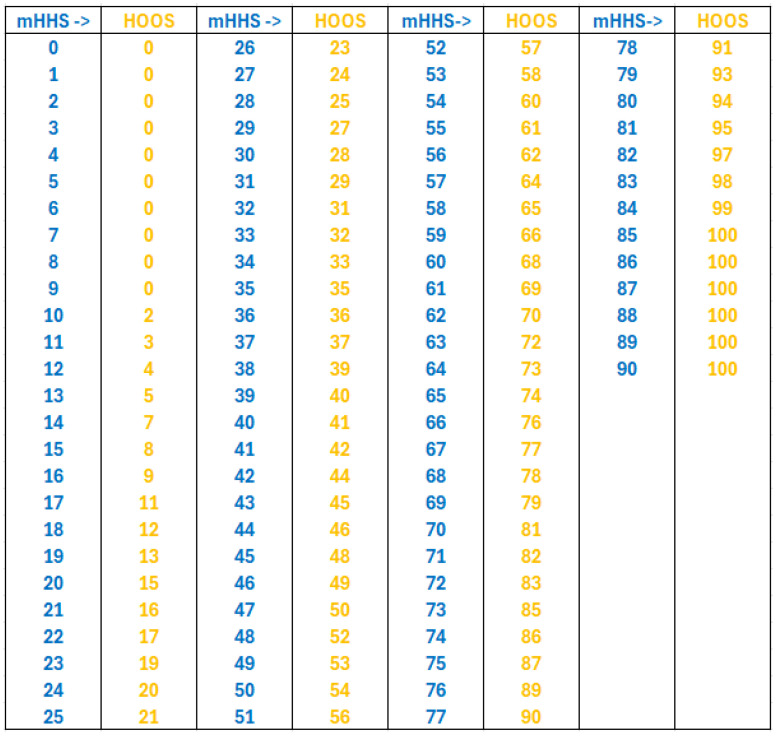
mHHS-to-HOOS crosswalk made by the Linear Regression Method (LR).

**Figure A7:**
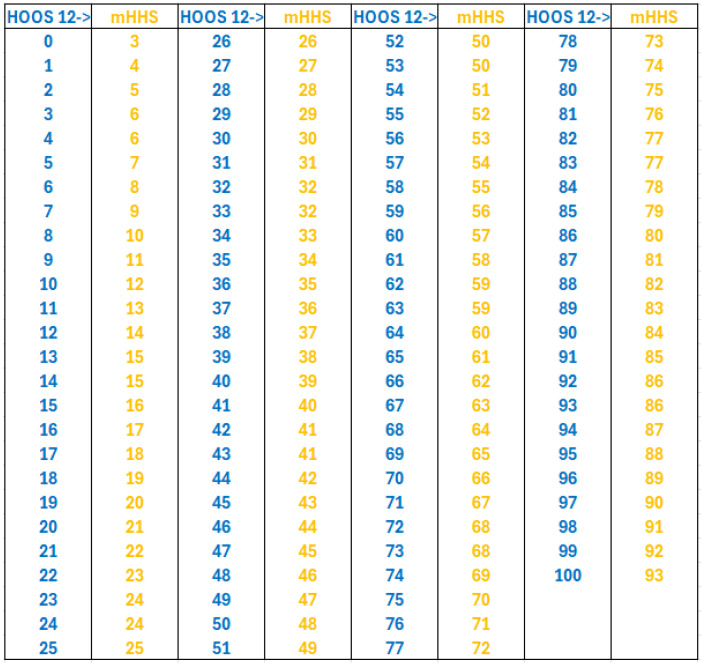
HOOS-12-to-mHHS crosswalk made by the Linear Regression Method (LR).

**Figure A8:**
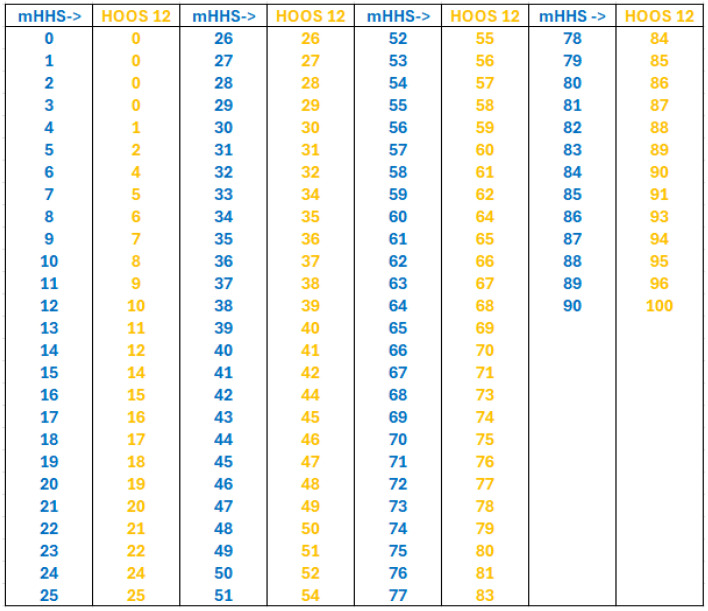
mHHS-to-HOOS-12 crosswalk made by the Linear Regression Method (LR).

There was a strong linear relationship between the PD mHHS and PD HOOS (R = 0.90, *p* < 0.0001). A similar result was found between the PD mHHS and PD HOOS-12 (R = 0.90, *p* < 0.0001). The linear relationships were greater than the R = 0.3 threshold considered acceptable to create a crosswalk defined by Dorans [27]. The assumptions for performing the EQ and LR crosswalks were met, and a total of eight crosswalks were created (see Appendix A, Figure A1, Figure A2, Figure A3, Figure A4, Figure A5, Figure A6, Figure A7 and Figure A8).

The overall MAE of the crosswalks, made using the EQ method, ranged between 8.2–10.4 (Table 1). The largest error of the EQ crosswalks was found at the mHHS-to-HOOS crosswalk at 3 months (12.3). The smallest error of the EQ crosswalks was identified at pre-op for the HOOS-to-mHHS crosswalk (6.7). The pre-op data generally contributed the least towards the error of the crosswalk for all four crosswalks and, in contrast, 3-month data contributed the most towards the error of the crosswalk.

**Figure 2:**
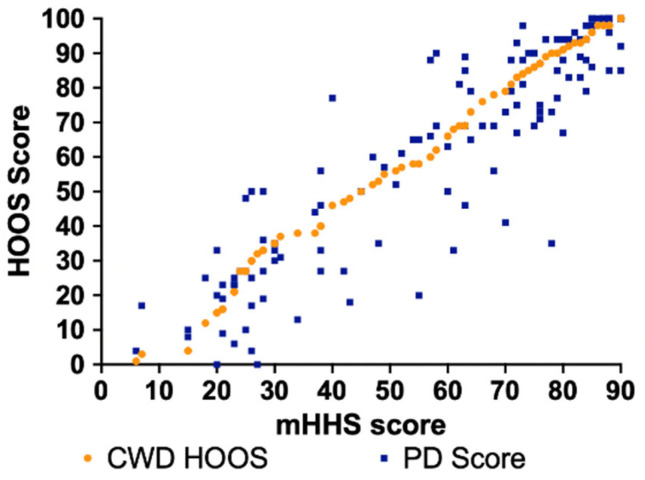
CWD and PD HOOS scores compared to PD mHHS using the EQ method.

**Figure 3:**
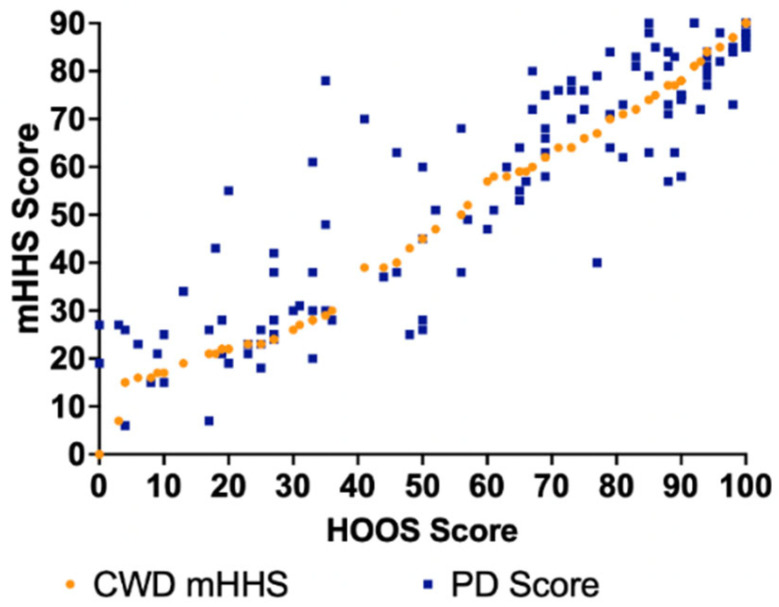
CWD and PD mHHS scores compared to PD HOOS using the EQ method.

The error for the EQ method was more evident in mHHS scores between 20 and 80 for both the HOOS-to-mHHS crosswalk and mHHS-to-HOOS crosswalk (Figure 2 and Figure 3). The largest differences generally occurred within this region as there were fewer data points/patients scoring in that range. The largest difference between the CWD HOOS and PD score (43 points) of the HOOS-to-mHHS crosswalk was found at mHHS = 35 (Figure 2). The largest difference between the CWD mHHS and PD score (43 points) of the mHHS-to-HOOS crosswalk was found at HOOS = 35 (Figure 3).

**Figure 4:**
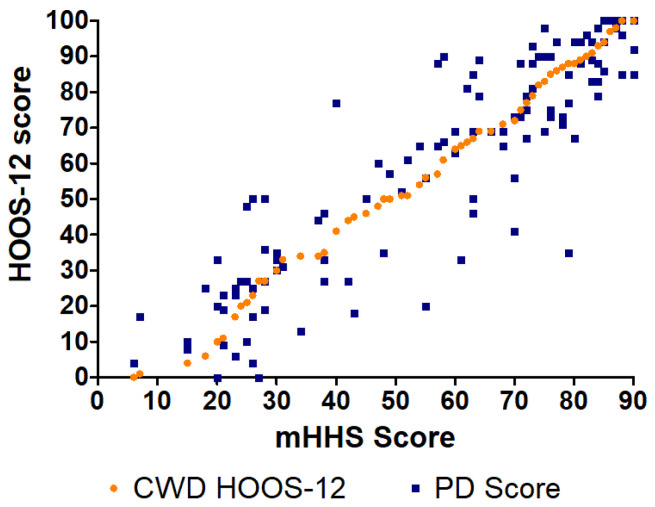
CWD and PD HOOS-12 scores compared to PD mHHS using the EQ method.

**Figure 5:**
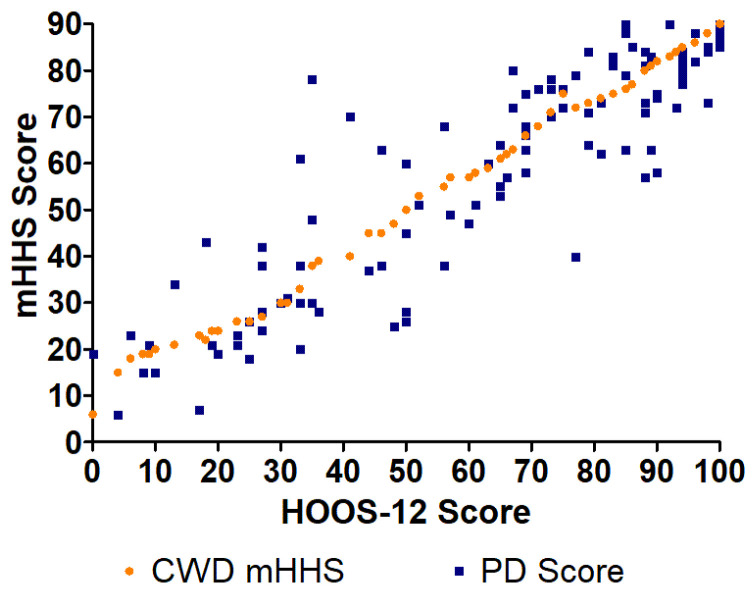
CWD and PD mHHS scores compared to PD HOOS-12 using the EQ method.

The largest difference between the CWD HOOS-12 and PD score (44 points) of the HOOS-12-to-mHHS crosswalk was found at mHHS = 79 (Figure 4). The largest difference between the CWD mHHS and PD score (43 points) of the mHHS-to-HOOS-12 crosswalk was found at HOOS-12 = 35 (Figure 5).

The LR method resulted in the following four equations to create crosswalks:

CWD mHHS = 1.0713 (PDHOOS) + 2.6526

CWD HOOS = 0.7568 (PDmHHS) + 8.8474

CWD mHHS = 1.1124 (PDHOOS-12) − 3.1421

CWD HOOS-12 = 0.728 (PDmHHS) + 13.192

The overall MAE of the crosswalks, made using the LR method, ranged between 8.4–10.4. The largest error of the LR crosswalk was found at the mHHS-to-HOOS crosswalk at 3 months (12.6) (Table 2). The smallest error of the LR crosswalk was identified at pre-op for the HOOS-to-mHHS crosswalk (7.2). The pre-op data generally contributed the least towards the error of the crosswalk for all four crosswalks, and in contrast, 3-month data contributed the most towards the error of the crosswalk. This pattern is similar to the errors identified in Table 1 for the EQ method.

**Figure 6:**
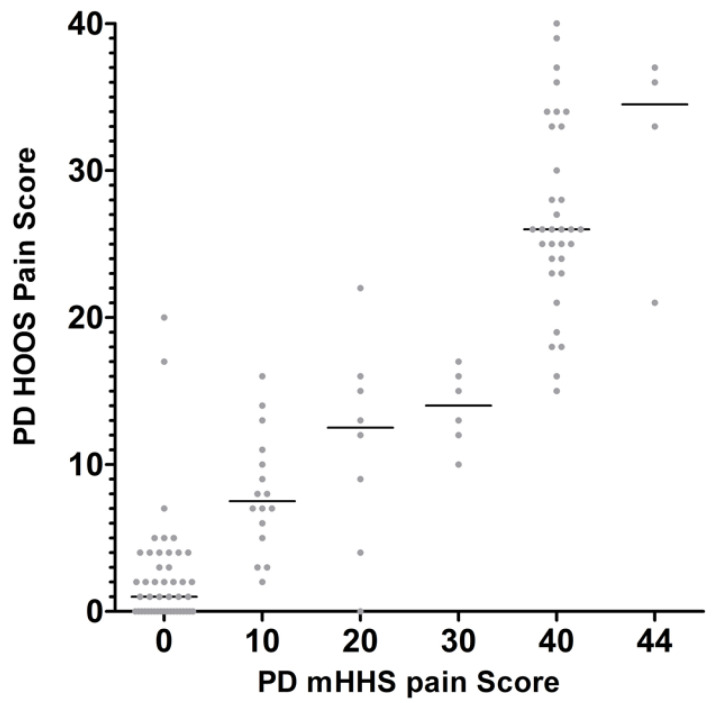
Each PD HOOS pain score matched to the equivalent PD mHHS pain score at the same time points.

**Figure 7:**
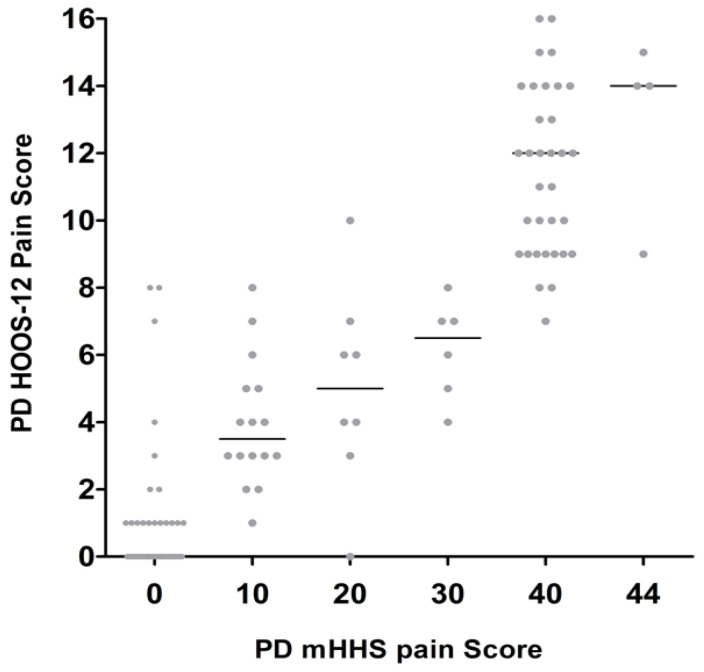
Each PD HOOS-12 pain score matched to equivalent PD mHHS pain score at the same time points.

The largest difference in PD HOOS pain scores was 25 at mHHS = 10 (Figure 6). The largest difference in the PD HOOS-12 pain score was 9 at mHHS = 10 (Figure 7).

**Figure 8:**
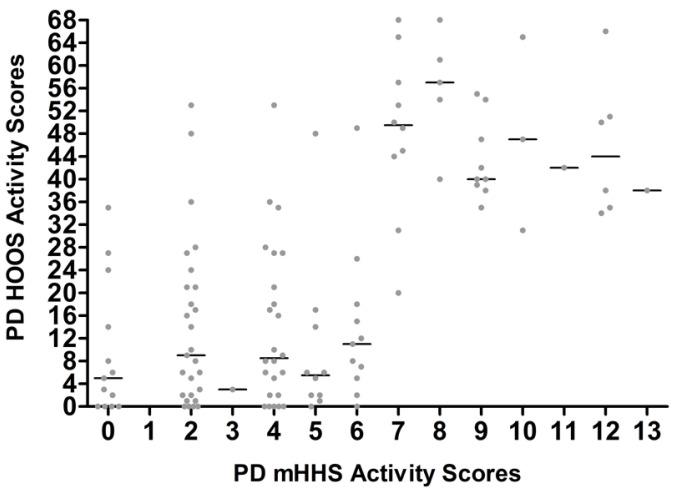
Each PD HOOS activity score matched to equivalent PD mHHS activity score at the same time points.

**Figure 9:**
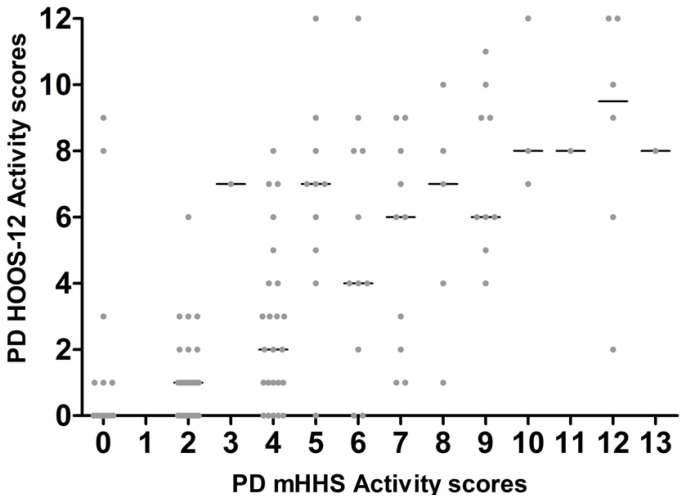
Each PD HOOS-12 activity score matched to equivalent PD mHHS activity score at the same time points.

There was a large variability seen between the PD mHHS activity and HOOS (Figure 8) and HOOS-12 activity (Figure 9).

Out of the two subcategories, differences in PD-reported activity had a larger contribution towards the error in all crosswalks than pain (Table 3).

## 4. Discussion

With the increased implant longevity following THA surgery now exceeding 2 decades, long-term PROM collections use crosswalks to convert older scores to new scoring methods. A recent systematic review of all crosswalks available following arthroplasty surgeries [16] confirmed our study to be the first to create a multi-directional crosswalk between mHHS, HOOS, and HOOS-12. Since mHHS has been the most widely used instrument across many orthopaedic institutions for over 5 decades, creating a crosswalk to newer measures such as the HOOS and HOOS-12, which are now incorporated within large International Registry collections, is beneficial.

The Spearman coefficients exceeded the acceptable threshold of 0.3, indicating that these crosswalks were able to be created and validated. The crosswalks will allow the conversion of HOOS and HOOS-12 scores to mHHS and vice versa, allowing either PROM to be used to assess patient outcomes following surgery. This is beneficial as the mHHS PROMs that have been collected in long-term THA studies can be used to compare directly with HOOS and HOOS-12 PROMs data in the future.

The MAE found between CWD scores and PD scores may, in part, be due to discrepancies in answers completed by patients for both the mHHS and HOOS on the same day. For example, one patient reported severe pain in the mHHS and only mild pain in the HOOS questionnaire at the same time point. The different wording of questions between the two PROMs will create inconsistent reporting. In particular, discrepancies were seen in the PD scores for HOOS and mHHS pain and activity, with larger variability observed for activity. For example, on the same day, one patient reported an activity score of 2 out of 13 on the mHHS, suggesting low functionality, while reporting a score of 53 out of 68 on the HOOS, which corresponds to good functionality. Conversely, one patient reported an activity score of 12 on the mHHS, suggesting excellent functionality, while scoring 37 on the HOOS, which corresponds to moderate-to-low functionality. Differences may arise because the HOOS emphasizes specific difficulties in daily activities, potentially capturing limitations that patients may not consider significant when answering the more general questions in the mHHS. The mHHS was developed to be used primarily for THA patients, whereas in contrast, the HOOS was developed with many more specific questions on pain and activities of daily living, with the aim to measure outcomes in other hip conditions and in younger patients. Subcategory analysis of the pain scores had less variability than activity. The variation in the pain scores is likely due to the more detailed 10 pain questions within HOOS aimed to capture the intensity of pain and the impact on specific activities. The mHHS evaluates pain more broadly in only one question, which may be influenced by other sites of pain, potentially leading to underreporting or overreporting. The one pain question in the mHHS, with six response options, results in larger increments between the patient response, which subsequently influences the overall score as pain constitutes 44 of the 90 total points. Therefore, the patient’s interpretation of the questions may contribute to the error of the crosswalks, particularly subcategories of activity and pain. Undertaking subcategory analysis was limited to domains of activity and pain as the question variability made it difficult to match other specific subcategories between mHHS and HOOS.

Despite the overall MAE ranging from 8.2 to 10.4, the crosswalks can still be used to identify patient improvement over time. For example, the minimal clinically important improvement threshold for the HHS is 16–18 points [29]. As the error of our crosswalks is below the threshold, we would be able to evaluate whether the patient had made improvement post-operation. Similarly, Lyman et.al reported a minimum clinically important difference between 13 and 36 points for the HOOS used in THA patients [30]. The error of our crosswalks was also below this threshold, and by using the mHHS-to-HOOS crosswalk, clinicians will be able to identify whether the patient has made clinically meaningful improvements by identifying the change in scores over time.

Studies have shown that PROMs are different at pre-op and post-operative intervals [31,32]. When the errors of our crosswalks were evaluated across time, it was found out that the 3-month postoperative data had the largest MAE, whilst the pre-op data had the smallest error when both the EQ and LR methods were used. This variation of the error could be occurring due to patients generally reporting more definitive lower scores pre-operatively while in pain, whilst at 3 months post-surgery the scores are more variable due to some patients not having fully recovered from their surgery, while others may have made full recovery. Previous studies have found PROMs to plateau after 6 months [31] and, hence, will result in more consistent scores thereafter. Except for one study, which investigated the WOMAC and EQ-5D in a hip and knee arthroplasty Spanish population [33], most crosswalk studies in the systematic review have used between 6 months and 2 years of follow-up data [16] when most patients have fully recovered. One of the strengths of this study is the inclusion of scores at the 3-month post-operative interval, a period when there is still variation in patient pain and functional-derived scores. Patient-derived scores are acknowledged to differ significantly at unique follow-up time points in an acute setting [31,32].

We used the EQ and LR methods to create our crosswalks and found similar errors of approximately 9%. The EQ method has been used in other studies that have developed crosswalks between HOOS and OHS or HOOS-12 and OHS 12 [17,22]. The advantage of using the EQ method over other methods is that it does not assume a linear relationship between the scores [28] and is relatively easy to use [34]. However, the EQ method requires that the PROM scales measure the same construct and have a Spearman coefficient greater than 0.3 between the PD scores of the two PROMs [17,22,27]. Therefore, the EQ method may not be suitable for creating other crosswalks with different PROM instruments and scores.

It was possible to create a crosswalk of the mHHS to the HOOS-12. The HOOS-12 has been established as a reliable THA outcome measure [13], but patients are only required to complete 12 questions instead of 40 questions in the original HOOS. This results in only three questions instead of ten for subcategory pain. Notably, we found that even with the fewer pain and activity questions within the HOOS-12, there was a similar distribution of the MAE in the multidirectional HOOS-12 crosswalks as there were in the HOOS crosswalks. Implementing the use of HOOS-12 instead of the HOOS can reduce the patient burden by significantly reducing the time taken and increasing the likelihood of questionnaire completeness.

There were several limitations of this study. The main limitation was that the crosswalk could not be directly created between HHS, HOOS, and HOOS-12. The HHS has a doctor-reported range of motion domain, which prevented the HHS from being classified as a PROM. However, by excluding the range of motion and public transport components of the HHS, we were able to use the mHHS as a self-reporting PROM. Secondly, our study included 49 patients and 121 matched PD scores to create the crosswalks. Although this number may be considered relatively small, Spearman’s coefficient of 0.9 showed a strong positive association between the PD mHHS and HOOS scores and was above the 0.3 threshold for creating crosswalks. Other crosswalks have been created with as little as 105 pre-operative PD scores [35], and our sample size calculation suggested that 67 matched PD scores were required. Due to the differences observed in PD-matched scores, we expect the MAE will not improve with more PD scores. Thirdly, conducting a study on a larger group in the future, with a more diverse population could improve the generalizability of the results since the mHHS or HOOS are collected on patients with different pathologies. Performing an external validation using data from other centres would be valuable, but mHHS and HOOS scores are not often prospectively collected on the same individuals at the same time points. Finally, only two different methodologies were used to create the crosswalks, and it was inconclusive as to whether the EQ method or LR method was the most appropriate way to develop the crosswalks. In a similar previous study that created PROM crosswalks using five different methods, the EQ method was found to be the most appropriate in creating crosswalks between two hip PROMs [17]. Since this is the first crosswalk to be created using mHHS and HOOS data, other methodologies, including Baysesian or, perhaps, machine learning could be used in the future to further assess the relationship between these two PROMs.

## 5. Conclusions

This is the first study to create a multi-directional crosswalk between the mHHS and HOOS, as well as mHHS and HOOS-12. Our crosswalks can be used to compare the outcome of THA in prospective studies while using older PROM collections as a control or quality-assurance benchmark. The use of the shorter HOOS-12 in future studies will reduce the patient burden of questionnaire completion.

## Figures and Tables

**Table 1:** Mean Absolute Error of four crosswalk-predicted scores relative to matching PD scores at each time point using the Equipercentile Method. The MAE should be interpreted relative to the denominator of the predicted PROM (HOOS 100; mHHS 90).

	mHHS to HOOS	HOOS to mHHS	mHHS to HOOS-12	HOOS-12 to mHHS
Overall	10.3	8.5	10.4	8.2
Pre–OP	9.8	6.7	9.7	7.3
3 Months	12.3	10.5	12.2	9.9
6 Months	9.2	7.9	9.2	7.2
12 Months	9.5	9.2	10.4	8.7

**Table 2:** Mean Absolute Error of four crosswalk-predicted scores relative to matching PD scores at each time point using the Linear Regression method. The MAE should be interpreted relative to the denominator of the predicted PROM (HOOS 100; mHHS 90).

	mHHS to HOOS	HOOS to mHHS	mHHS to HOOS-12	HOOS-12 to mHHS
Overall	10.4	8.4	10.1	9.0
Pre–OP	9.4	7.2	8.7	7.9
3 Months	12.6	10.1	12.0	10.5
6 Months	9.3	7.6	9.5	8.1
12 Months	10.4	8.6	11.0	9.4

**Table 3:** Mean Absolute Error of the subcategory pain and activity scores of the four crosswalks made by the Equipercentile Method. The MAE should be interpreted relative to the denominator of each sub-category (HOOS pain 100; HOOS activity 100; mHHS pain 44; mHHS activity 13).

Subcategory	mHHS to HOOS	HOOS to mHHS	mHHS to HOOS-12	HOOS-12 to mHHS
Pain	12.9	7.0	16.7	4.2
Activity	21.1	2.4	18.9	2.4

## Data Availability

The data presented in this study are available on request from the corresponding author due to privacy and ethical restrictions.
